# Association of stroke unit certification with process quality in acute ischemic stroke: a registry-based study

**DOI:** 10.1186/s42466-026-00532-1

**Published:** 2026-09-25

**Authors:** Malin Boehl, Max Geraedts, Limei Ji, Björn Misselwitz, Tobias Neumann-Haefelin

**Affiliations:** 1 Department of Neurology, Universitätsmedizin Marburg - Campus Fulda, Fulda, Germany; 2 Institute for Health Services Research and Clinical Epidemiology, School of Medicine, Marburg University, Marburg, Germany; 3 Institute of Quality Assurance Hesse, Eschborn, Germany; 4 Department of Neurology, Klinikum Fulda, Pacelliallee 4, 36043 Fulda, Germany

**Keywords:** Ischemic stroke, Intravenous thrombolysis, Stroke unit certification, Process quality, Door-to-needle time, Hyperacute stroke care

## Abstract

**Background:**

Certification of primary and comprehensive Stroke Units (pSU, cSU) was established in Germany to ensure standardized, guideline-adherent and quality-assured care for patients with acute ischemic stroke. However, real-world evidence remains limited that treatment quality is different in certified institutions. In this analysis we focused on potential differences between certified and non-certified institutions concerning various aspects of intravenous thrombolysis.

**Methods:**

We conducted a retrospective registry-based observational analysis of patients with acute ischemic stroke in Hesse, Germany, between 2021 and 2024. Associations between stroke unit certification, intravenous thrombolysis (IVT), and process quality indicators were assessed using multivariable generalized linear mixed-effects models accounting for hospital-level clustering.

**Results:**

A total of 59,514 patients with acute ischemic stroke were included. IVT within 4.5 h after symptom onset was performed significantly more often in certified stroke units than in non-certified hospitals (pSUs: OR 2.32, 95% CI 1.65–3.27; cSUs: OR 2.19, 95% CI 1.57–3.07). In the extended treatment window (> 4.5–24 h), higher odds of IVT were also observed in pSUs (OR 2.41, 95% CI 1.16–5.00) and cSUs (OR 4.20, 95% CI 2.07–8.50). Certified stroke units achieved guideline-recommended targets more often, including a door-to-needle time ≤ 60 min (pSUs: OR 3.13, 95% CI 2.13–4.61; cSUs: OR 2.60, 95% CI 1.80–3.75) and ≤ 30 min (pSUs: OR 1.85, 95% CI 1.04–3.28; cSUs: OR 2.84, 95% CI 1.62–4.99). No significant differences were observed between pSUs and cSUs.

**Conclusions:**

Stroke unit certification was associated with a more frequent use of IVT and shorter door-to-needle times.

**Supplementary Information:**

The online version contains supplementary material available at https://doi.org/10.1186/s42466-026-00532-1.

## Background

Stroke remains one of the leading causes of death and long-term disability worldwide and represents a major burden on healthcare systems [1, 2]. Rapid access to evidence-based acute stroke treatment is critical, as functional outcomes are strongly influenced by timely reperfusion therapy and efficient hyperacute stroke care pathways [3–6].

Intravenous thrombolysis is an evidence-based treatment for acute ischemic stroke and has been consistently associated with improved functional outcomes when administered rapidly after symptom onset [3, 7, 8].

Because treatment benefit declines with increasing delays, optimization of hyperacute stroke workflows and minimization of door-to-needle times have become key priorities in contemporary stroke care. Consequently, door-to-needle time has emerged as one of the most important process quality indicators in acute ischemic stroke treatment [5, 9, 10].

Over the past decades, specialized stroke unit care has become a central component of modern stroke management and has been associated with improved clinical outcomes [11–15]. In Germany, a nationwide stroke unit certification system was established to promote standardized, guideline-adherent, and quality-assured stroke care [16, 17].

Certification is based on predefined structural, process-related, and quality assurance criteria, including the availability of specialized medical and nursing staff, neurological expertise, advanced imaging, and standardized acute stroke pathways. The system distinguishes between primary stroke units (pSUs), providing local acute stroke care including intravenous thrombolysis, and comprehensive stroke units (cSUs) with extended diagnostic and interventional capabilities, particularly for mechanical thrombectomy [16–19].

As of May 2026, 354 stroke units are certified in Germany [20]. Previous studies have demonstrated beneficial effects of structured quality assurance measures and specialized stroke care on the quality of stroke treatment and clinical outcomes [21–24]. Analyses from the German QUASCH project and other nationwide studies have shown that structure related quality indicators, external quality assurance, and treatment in specialized stroke units are associated with improved stroke care and lower mortality [21–23].

Similar findings have been reported from the United States, where higher-certified stroke units more frequently achieved guideline-recommended door-to-needle time targets compared with primary stroke units [25].

Nevertheless, evidence regarding the association between different certification levels and hyperacute stroke process quality metrics within the German stroke unit certification system remains limited.

Therefore, this study aimed to evaluate whether different stroke unit certification levels are associated with differences in IVT utilization and time-sensitive hyperacute stroke process metrics.

## Methods

### Data source

This retrospective registry-based study used data from the statutory external quality assurance stroke registry for the state of Hesse, Germany (LAGQH, Eschborn, Germany), collected between 2021 and 2024. Data entry is mandatory by federal law and clinical data are prospectively documented by the responsible treating physicians using standardized documentation forms. The registry covers more than 95% of all hospitalized stroke patients in Hesse, and data completeness is periodically verified against hospital administrative billing data. Documentation includes all hospitalized adult patients with acute ischemic stroke (ICD-10: I63.x) admitted within seven days after symptom onset. In approximately 5% of eligible cases, only a predefined minimal dataset is available, most commonly when stroke onset occurred more than seven days before hospital admission or when palliative treatment goals had already been established prior to admission.

Certified stroke units were further subdivided into pSUs (“regionale SU”) and cSUs (“überregionale SU”) according to the German stroke unit certification system [19]. One certified hospital belonged to the category of telemedicine-linked stroke units (Tele-SU). These cases were included in analyses comparing certified versus non-certified hospitals but were excluded from analyses stratified by certification level because Tele-SUs cannot be assigned to either the pSU or cSU category.

### Outcome

The study focused on intravenous thrombolysis (IVT) and associated process parameters. The analysis included the following parameters: overall IVT rate, IVT rate within the conventional (≤ 4.5 h) and extended (> 4.5–24 h) time window, overall door-to-needle time (DTN) and rates of DTN ≤ 60 and ≤ 30 min. The selected process quality metrics reflect established guideline-based quality indicators. For the conventional treatment-window analysis (≤ 4.5 h), hospital admission within ≤ 4 h after symptom onset was required to allow sufficient time for in-hospital evaluation and initiation of IVT within the treatment window. Accordingly, for the extended treatment-window analysis (> 4.5–24 h), patients admitted > 4 h and up to 24 h after symptom onset were considered. Detailed definitions of the study populations used for each analysis are provided in Supplementary Table S1.

### Statistical analyses

Categorical variables were compared using chi-square tests and continuous variables using Kruskal–Wallis tests. Associations between certification status and study outcomes were assessed using multivariable generalized linear mixed-effects models with a binomial distribution and logit link. To account for clustering of patients within hospitals, hospital site was included as a random intercept. Depending on the outcome analyzed, adjustment variables included age, sex, admission NIHSS score, vascular risk factors, anticoagulation status, and interhospital transfer status where applicable. The exact covariates and the final sample sizes for each outcome-specific model are provided in Supplementary Table S1. All variables included in the analyses were mandatory fields in the quality assurance dataset; therefore, no missing-data imputation was required. No convergence warnings were observed for the fitted mixed-effects models. Multicollinearity among covariates was assessed using variance inflation factors (VIFs), with all VIFs < 2. Results are reported as adjusted odds ratios (ORs) with 95% confidence intervals (CIs). A two-sided p-value < 0.05 was considered statistically significant.

## Results

### Hospital distribution

Between 2021 and 2024, a total of 59,514 patients with acute ischemic stroke were included. Overall, 9,819 patients were treated in 62 non-certified hospitals, 17,940 in 11 pSUs, 31,056 in 12 cSUs, and 699 in one Tele-SU. Baseline characteristics are shown in Table 1.

Patients treated in cSUs had higher stroke severity at admission, as reflected by higher median NIHSS scores, whereas age and sex distributions were largely comparable across certification levels.

**Table 1:** Baseline characteristics of the study population according to certification level

Characteristic	Non-certified hospitals (*n* = 9,819)	pSUs (*n* = 17,940)	cSUs (*n* = 31,056)	*p*-value
Age, years, median (IQR) ᵃᶜ	77 (67–84)	76 (66–84)	75 (64–83)	< 0.001
**Sex**,** n (%) ᵇ**				0.104
Male	5,229 (53.25)	9,588 (53.42)	16,846 (54.24)	
Female	4,590 (46.75)	8,352 (46.56)	14,210 (45.76)	
**Comorbidities**,** n (%) ᵇᵈ**				
Diabetes mellitus	2,788 (29.55)	4,857 (28.29)	8,051 (26.30)	< 0.001
Atrial fibrillation	2,491 (26.40)	4,442 (25.87)	8,790 (28.72)	< 0.001
Previous stroke	2,387 (25.30)	4,151 (24.18)	6,754 (22.07)	< 0.001
Hypertension	7,703 (81.64)	14,010 (81.60)	24,787 (80.98)	0.155
**Clinical parameters at admission**				
NIHSS, median (IQR) ᵃᵉ	3 (1–6)	3 (1–6)	4 (2–9)	< 0.001
Anticoagulation, n (%) ᵇ	1,944 (19.80)	3,238 (18.05)	5,497 (17.70)	< 0.001

ᵃ Kruskal–Wallis test for continuous variables

ᵇ Chi-square test for categorical variables

ᶜ Available cases for age: non-certified hospitals, *n* = 9,283; pSUs, *n* = 17,717; cSUs, *n* = 31,024

ᵈ Available cases for comorbidity variables: non-certified hospitals, *n* = 9,345; pSUs, *n* = 17,170; cSUs, *n* = 30,608

ᵉ Available cases for NIHSS: non-certified hospitals, *n* = 9,283; pSUs, *n* = 17,717; cSUs, *n* = 31,024

IQR, interquartile range; NIHSS, National Institutes of Health Stroke Scale; pSU, primary stroke unit; cSU, comprehensive stroke unit

### Intravenous thrombolysis utilization

Overall, intravenous thrombolysis was administered in 19.0% of patients. IVT rates were 11.7% in non-certified hospitals, 19.0% in pSUs, and 21.4% in cSUs (*p* < 0.001).

Multivariable generalized linear mixed-effects models showed that IVT was performed more often in both pSUs and cSUs compared with non-certified hospitals (pSU: OR 2.15, 95% CI 1.56–2.96; cSU: OR 2.31, 95% CI 1.69–3.16; both *p* < 0.001). No statistically significant difference in IVT utilization was observed between cSUs and pSUs (OR 1.08, 95% CI 0.76–1.53; *p* = 0.676).

In the conventional treatment-window analysis (≤ 4.5 h), IVT was administered in 41.6% of patients in non-certified hospitals, 55.6% in pSUs, and 58.3% in cSUs (*p* < 0.001). Both pSUs and cSUs demonstrated significantly higher odds of IVT administration compared with non-certified hospitals (pSU: OR 2.32, 95% CI 1.65–3.27; cSU: OR 2.19, 95% CI 1.57–3.07; both *p* < 0.001), whereas no significant difference was found between pSUs and cSUs (OR 0.95, 95% CI 0.67–1.34; *p* = 0.751).

In the extended treatment-window analysis (> 4.5–24 h), IVT was administered in 2.1% of patients in non-certified hospitals, 4.9% in pSUs, and 8.3% in cSUs (*p* < 0.001). Compared with non-certified hospitals, both pSUs and cSUs showed significantly increased odds of thrombolysis (pSU: OR 2.41, 95% CI 1.16–5.00; *p* = 0.018; cSU: OR 4.20, 95% CI 2.07–8.50; *p* < 0.001). However, no significant difference was found between cSUs and pSUs (OR 1.74, 95% CI 0.89–3.42; *p* = 0.108).

Patients with unknown symptom onset, including wake-up strokes, were not included in our principal analysis. However, in a separate wake-up stroke analysis, a similar pattern was observed, with IVT rates of 10.7% in non-certified hospitals, 19.3% in pSUs, and 28.4% in cSUs (*p* < 0.001). Compared with non-certified hospitals, the association was significant for cSUs (OR 3.18, 95% CI 1.57–6.45; *p* = 0.001), but not for pSUs (OR 1.88, 95% CI 0.90–3.92; *p* = 0.094). No significant difference was found between cSUs and pSUs (OR 1.70, 95% CI 0.93–3.08; *p* = 0.084).

**Fig. 1:**
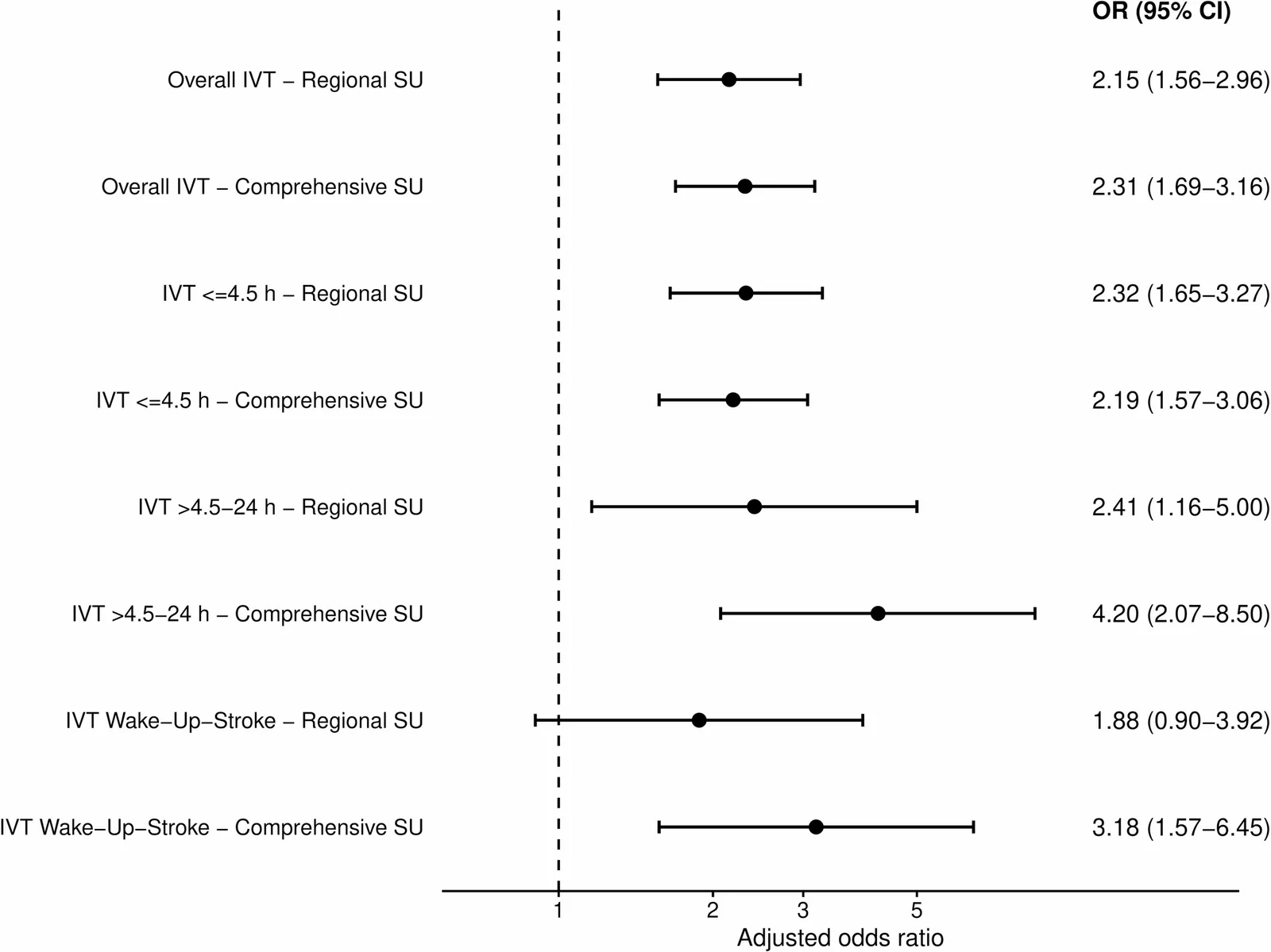
Association between stroke unit certification level and intravenous thrombolysis utilization. Adjusted odds ratios (ORs) with 95% confidence intervals derived from multivariable generalized linear mixed-effects models comparing pSUs and cSUs with non-certified hospitals. Hospital site was included as a random intercept to account for clustering of patients within hospitals. Error bars indicate 95% confidence intervals. The vertical dashed line represents the reference value (non-certified hospitals, OR = 1)

**Fig. 2:**
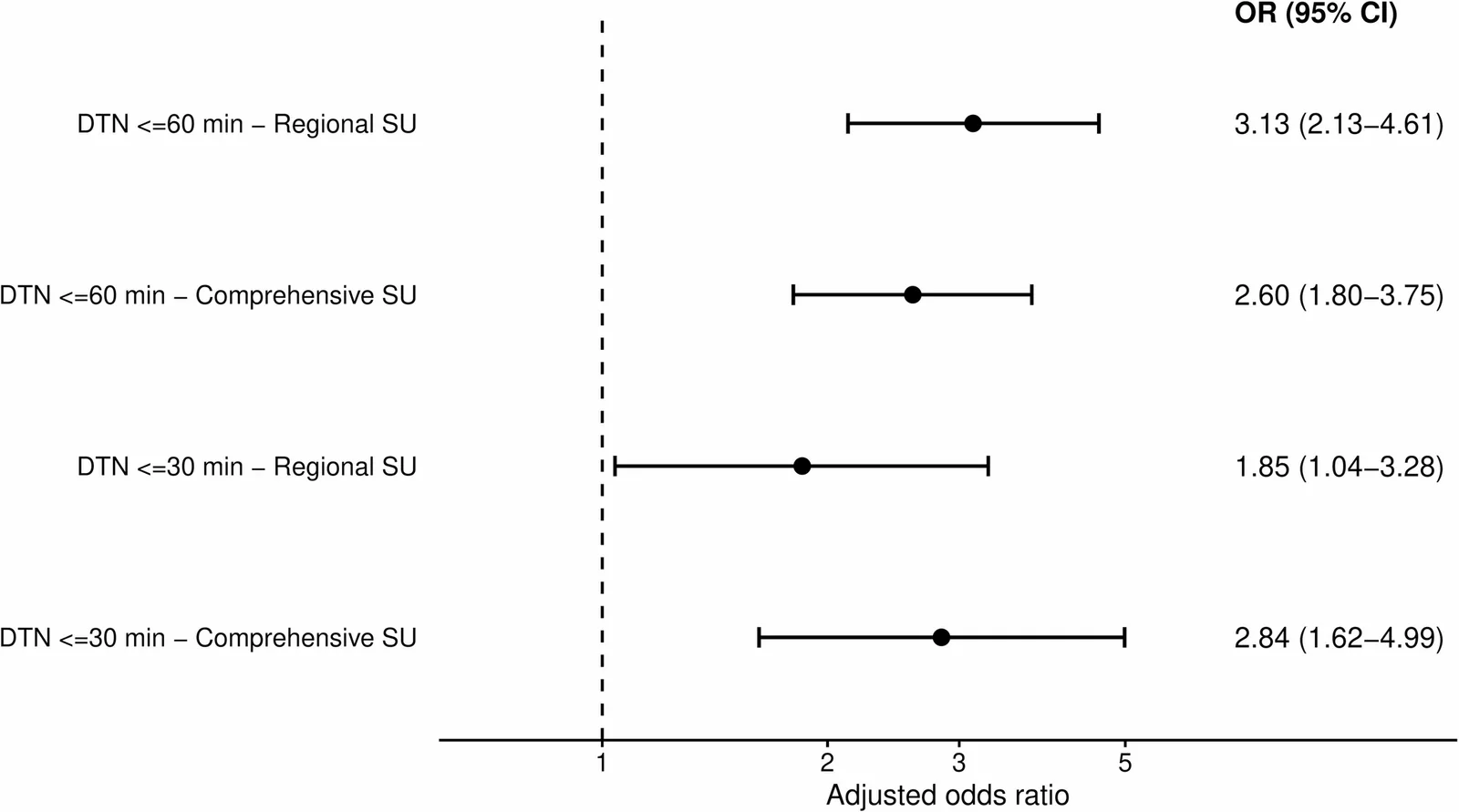
Association between stroke unit certification level and door-to-needle time targets. Adjusted odds ratios (ORs) with 95% confidence intervals derived from multivariable generalized linear mixed-effects models comparing pSUs and cSUs with non-certified hospitals. Hospital site was included as a random intercept to account for clustering of patients within hospitals. Error bars indicate 95% confidence intervals. The vertical dashed line represents the reference value (non-certified hospitals, OR = 1)

**Fig. 3:**
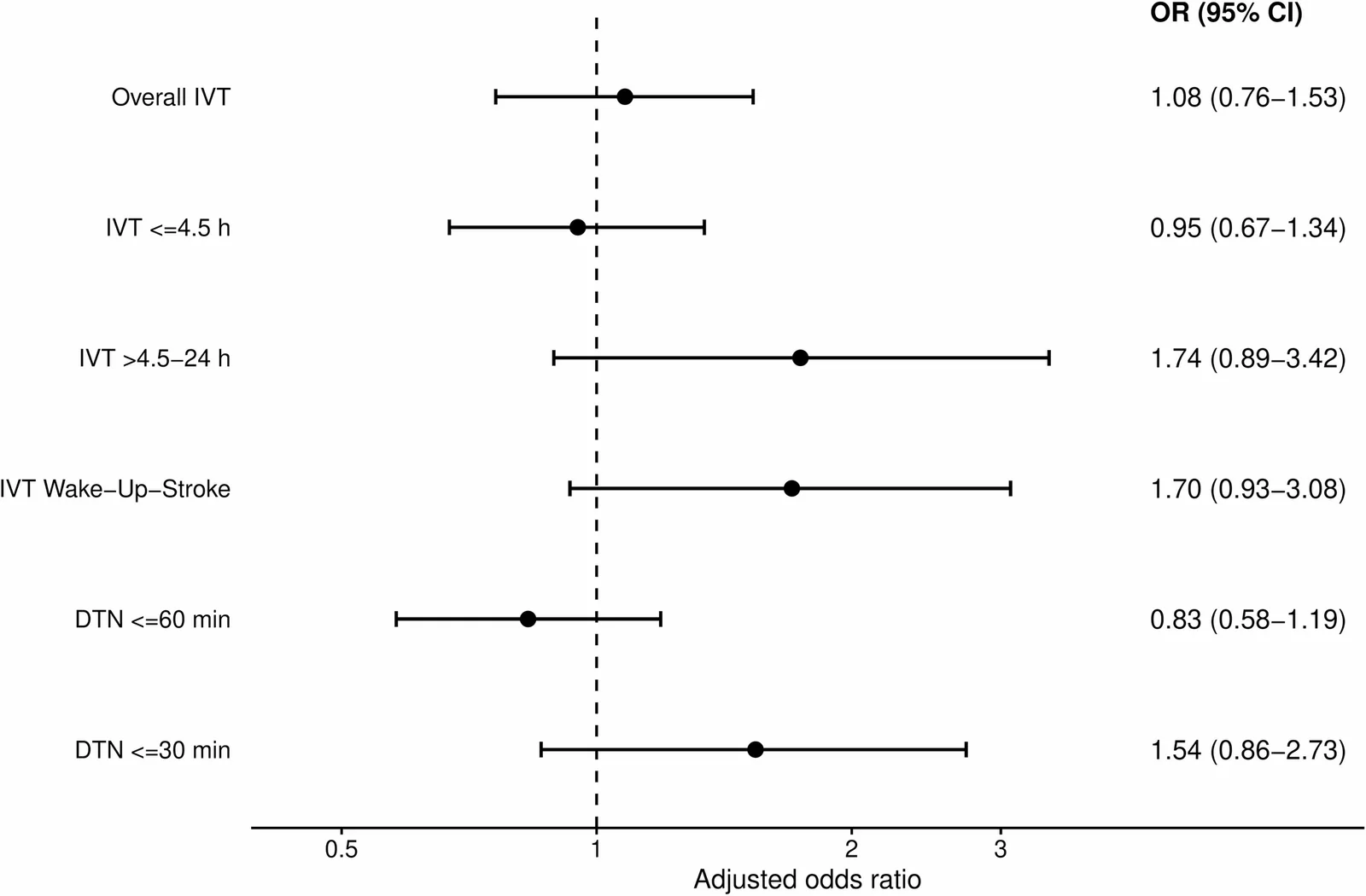
Association of cSU versus pSU certification with intravenous thrombolysis utilization and door-to-needle time metrics. Adjusted odds ratios (ORs) with 95% confidence intervals derived from multivariable generalized linear mixed-effects models. Hospital site was included as a random intercept to account for clustering of patients within hospitals. Odds ratios greater than 1 indicate higher odds in cSUs compared with pSUs. Error bars indicate 95% confidence intervals. The vertical dashed line represents the reference category (pSUs, OR = 1)

Table 2 summarizes all adjusted mixed-effects regression analyses, while the corresponding adjusted odds ratios and 95% confidence intervals are displayed in Figs. 1, 2 and 3.

**Table 2:** Adjusted mixed-effects regression analyses according to stroke unit certification status

Outcome	pSU vs. non-certified OR (95% CI) ᵃ ᶜ	*p*-value	cSU vs. non-certified OR (95% CI) ᵃ ᶜ	*p*-value	cSU vs. pSU OR (95% CI) ᵇ ᶜ	*p*-value
IVT utilization	2.15 (1.56–2.96)	< 0.001	2.31 (1.69–3.16)	< 0.001	1.08 (0.76–1.53)	0.676
IVT ≤ 4.5 h	2.32 (1.65–3.27)	< 0.001	2.19 (1.57–3.07)	< 0.001	0.95 (0.67–1.34)	0.751
IVT > 4.5–24 h	2.41 (1.16–5.00)	0.018	4.20 (2.07–8.50)	< 0.001	1.74 (0.89–3.42)	0.108
IVT Wake-Up-Stroke	1.88 (0.90–3.92)	0.094	3.18 (1.57–6.45)	0.001	1.70 (0.93–3.08)	0.084
DTN ≤ 60 min	3.13 (2.13–4.61)	< 0.001	2.60 (1.80–3.75)	< 0.001	0.83 (0.58–1.19)	0.311
DTN ≤ 30 min	1.85 (1.04–3.28)	0.037	2.84 (1.62–4.99)	< 0.001	1.54 (0.86–2.73)	0.143

ᵃ Reference category: non-certified hospitals

ᵇ Reference category: pSU

ᶜ Odds ratios were derived from multivariable generalized linear mixed-effects models with hospital site included as a random intercept to account for clustering of patients within hospitals. Models were adjusted for age, sex, admission NIHSS score, diabetes mellitus, atrial fibrillation, previous stroke, anticoagulation, and interhospital transfer status, where applicable

OR, odds ratio; CI, confidence interval; IVT, intravenous thrombolysis; DTN, door-to-needle time; pSU, primary stroke unit; cSU, comprehensive stroke unit

### Time-to-treatment

The median door-to-needle time was 32 min overall, with shorter treatment times in certified stroke units than in non-certified hospitals. Median DTN was 39 min in non-certified hospitals, 33 min in pSUs, and 30 min in cSUs.

Certified stroke units more frequently achieved predefined DTN targets compared with non-certified hospitals. DTN ≤ 60 min was achieved in 80.9% of patients in non-certified hospitals, in 91.8% in pSUs, and in 89.9% in cSUs (*p* < 0.001). In the multivariable analysis, both pSUs and cSUs were associated with higher odds of achieving guideline-recommended door-to-needle times ≤ 60 min (pSU: OR 3.13, 95% CI 2.13–4.61; cSU: OR 2.60, 95% CI 1.80–3.75; both *p* < 0.001). No significant difference was found between pSUs and cSUs in achieving this target (OR 0.83, 95% CI 0.58–1.19; *p* = 0.311).

DTN ≤ 30 min was achieved in 37.6% of patients in non-certified hospitals, 45.1% in pSUs, and 53.6% in cSUs (*p* < 0.001). Despite the numerically highest rate in cSUs, no significant difference was found between cSUs and pSUs in the adjusted analysis (OR 1.54, 95% CI 0.86–2.73; *p* = 0.143). Compared with non-certified hospitals, both certification levels were associated with higher odds of achieving DTN ≤ 30 min (pSU: OR 1.85, 95% CI 1.04–3.28; *p* = 0.037; cSU: OR 2.84, 95% CI 1.62–4.99; *p* < 0.001).

## Discussion

In this large registry-based analysis of acute ischemic stroke care, stroke unit certification was independently associated with both a higher rate of patients receiving intravenous thrombolysis and treatment within predefined DTN targets. Notably, among patients admitted within the conventional time window for thrombolysis, more than half of those treated in certified stroke units received IVT.

When comparing pSUs and cSUs, no significant differences were observed regarding IVT within the conventional treatment window. Comprehensive SUs showed numerically higher rates of thrombolysis in the extended treatment window and in patients with wake-up stroke, as well as higher rates of achieving very short DTN times (≤ 30 min); however, these differences did not reach statistical significance in the adjusted mixed-effects analyses.

Although non-certified hospitals performed less favorably in direct comparison with certified SUs, a substantial proportion of patients treated in these hospitals still achieved very short DTN times (37.6% ≤30 min), suggesting that efficient hyperacute stroke processes may also be achievable outside formally certified SUs.

The observed differences between certified and non-certified hospitals are consistent with previous studies reporting associations of structured quality assurance systems and specialized stroke care with treatment quality and clinical outcomes [21–24]. While earlier studies primarily focused on clinical outcomes and general quality indicators, the present analysis found an association between stroke unit certification and favorable hyperacute stroke workflow performance, including thrombolysis utilization and door-to-needle time metrics. These findings are also consistent with previous observations from the United States, where differences in reperfusion care metrics were observed across levels of stroke center certification, without evidence of uniformly better performance with each higher certification level [25]. Although the US certification categories are not directly equivalent to German pSUs and cSUs, both systems distinguish between different levels of certified stroke care. Similarly, our findings suggest that higher certification level was not associated with a stepwise improvement in the investigated process metrics.

Several limitations should be considered when interpreting these findings. First, the retrospective observational design precludes causal inferences. Second, despite adjustment for clinically relevant covariates, residual confounding cannot be excluded. In particular, differences in referral patterns and case mix between non-certified hospitals, pSUs, and cSUs may have influenced the observed associations. Third, exact onset-to-IVT times were unavailable, and treatment windows were approximated based on onset-to-admission and DTN times; therefore, classification may not fully correspond to actual IVT treatment within or beyond 4.5 h. Fourth, the analysis was based on registry data from a single German federal state with a population of 6.2 million, which may limit the generalizability of the findings to other healthcare systems and stroke care settings. Fifth, the present analysis was limited to IVT utilization and DTN metrics and did not assess functional outcomes, treatment safety, or other aspects of overall stroke care quality. Nevertheless, the large real-world cohort, comprehensive statewide coverage, use of standardized external quality assurance data, and consideration of hospital-level clustering in the mixed-effects models represent important strengths of the present study.

## Conclusion

In the federal state of Hesse in Germany, most ischemic stroke patients are currently treated in certified SUs. However, there remains a substantial proportion of patients (16.7%) who are treated at non-certified hospitals. In certified SUs the rate of intravenous thrombolysis was higher, and DTN times were shorter. In addition, more patients were treated in the extended time window for thrombolysis, and very short DTN times (≤ 30 min) were achieved more frequently.

## Supplementary Information

Supplementary Material 1.

## Data Availability

No datasets were generated or analysed during the current study.
