# Supplementary material for: Association of stroke unit certification with process quality in acute ischemic stroke: a registry-based study
Source: Neurol Res Pract. 2026 Sep 25;8(1):79. doi: 10.1186/s42466-026-00532-1 (PMC13615603; doi:10.1186/s42466-026-00532-1)
Supplement: Supplementary file 1 [file 42466_2026_532_MOESM1_ESM.docx]

**Supplementary**

**Table S1:** Study populations used for the individual analyses

| **Analysis** | **Study population** | **Covariates included in adjusted model** | **Final model, n** |
| --- | --- | --- | --- |
| Overall IVT utilization | All patients with acute ischemic stroke. Outcome: intravenous thrombolysis performed in the treating hospital. | Age, sex, admission NIHSS, diabetes mellitus, previous stroke, hypertension, atrial fibrillation, anticoagulation, interhospital transfer | 56,432 |
| IVT within the conventional treatment window (≤4.5 h) **ᵃ** | Patients with symptom onset-to-admission time ≤4 h, including in-hospital strokes, allowing sufficient time for in-hospital evaluation and treatment within the 4.5h treatment window. Excluding patients transferred for thrombolysis, patients receiving thrombolysis at a referring hospital, patients with documented pre-stroke anticoagulation, and patients undergoing isolated mechanical thrombectomy without IVT. | Age, sex, admission NIHSS, diabetes mellitus, previous stroke, hypertension, atrial fibrillation | 13,603 |
| IVT within the extended treatment window (>4.5–24 h) **ᵃ** | Patients with symptom onset-to-admission time between >4 and 24 h. Excluding patients transferred for thrombolysis, patients receiving thrombolysis at a referring hospital, patients with documented pre-stroke anticoagulation, and patients undergoing isolated mechanical thrombectomy without IVT. | Age, sex, admission NIHSS, diabetes mellitus, previous stroke, hypertension, atrial fibrillation | 12,769 |
| IVT Wake-Up-Stroke | Patients with unknown symptom onset (Wake-Up-Stroke). Excluding patients transferred for thrombolysis, patients receiving thrombolysis at a referring hospital, patients with documented pre-stroke anticoagulation, and patients undergoing isolated mechanical thrombectomy without IVT. Wake-Up-Stroke data were available from 2022 onwards. | Age, sex, admission NIHSS, diabetes mellitus, previous stroke, hypertension, atrial fibrillation | 2,169 |
| Overall door-to-needle time (DTN) **ᵇ** | Patients receiving IVT in the treating hospital. In-hospital strokes excluded. Analyses restricted to documented DTN values between 0 and 120 min. ᶜ | **-** | **-** |
| DTN ≤60 min | Patients with acute ischemic stroke receiving IVT in the treating hospital and symptom onset-to-admission time ≤4 h. In-hospital strokes excluded. | Age, sex, admission NIHSS, diabetes mellitus, previous stroke, hypertension, atrial fibrillation, anticoagulation, interhospital transfer | 7,400 |
| DTN ≤30 min | Patients with acute ischemic stroke receiving IVT in the treating hospital and symptom onset-to-admission time ≤4 h. In-hospital strokes excluded. | Age, sex, admission NIHSS, diabetes mellitus, previous stroke, hypertension, atrial fibrillation, anticoagulation, interhospital transfer | 7,400 |

**ᵃ** As the exact time from symptom onset to IVT administration was not available, treatment windows were approximated using the documented symptom onset-to-admission time and door-to-needle time.

**ᵇ** Overall DTN was analyzed descriptively as a continuous variable and was not included in a multivariable mixed-effects model.

ᶜ DTN values >120 minutes were excluded as potentially implausible to reduce potential distortion of the process-time analyses.

**Abbreviations:** IVT, intravenous thrombolysis; DTN, door-to-needle time.
